# Rabies virus transmission via solid organs or tissue allotransplantation

**DOI:** 10.1186/s40249-018-0467-7

**Published:** 2018-08-15

**Authors:** Xue-Xin Lu, Wu-Yang Zhu, Gui-Zhen Wu

**Affiliations:** National Institute For Viral Disease Control and Prevention, Chinese Center For Disease Control and Prevention, 155 Changbai Road Changping District, Beijing, People’s Republic of China

**Keywords:** Rabies, Solid organ or tissue allotransplantation, Donor, Recipient, Zoonosis

## Abstract

**Background:**

Rabies, for which the mortality rate is almost 100%, is a zoonotic viral disease that can be transmitted via solid organs or tissue allotransplantation. Dozens of deaths from rabies via solid organs or tissues allotransplantation (ROTA) have been documented during the last decades. In 2015 and 2016, two cases of rabies virus transmission via solid organs or tissue allotransplantation were reported in China, which further underscore the risk and importance of this special type of rabies for organ transplant recipients.

**Main text:**

From 1978 to 2017, at least 13 cases of ROTA, causing dozens of deaths, have been reported worldwide, whether in the high-risk or low-risk countries of rabies. The reported incubation period of ROTA ranges from 11 days to more than 17 months, while the historical incubation period of rabies is generally considered to range from ~ 1 week to several years. The pathogenesis of ROTA is not clear, but the use of post-exposure prophylaxis (PEP) can play a protective role in the transplant recipients. We also summarize reports about ROTA in China, combined with the actual situation regarding work on rabies surveillance and elimination, and suggest countermeasures for the prevention and control of ROTA in the future.

**Conclusions:**

Understanding the significance of ROTA, screening the suspected organs, assessing the risk and protecting the related population will be effective way to prevent and control further occurrence of ROTA.

**Electronic supplementary material:**

The online version of this article (10.1186/s40249-018-0467-7) contains supplementary material, which is available to authorized users.

## Multilingual abstracts

Please see Additional file 1 for translations of the abstract into the five official working languages of the United Nations.

## Background

Since the 1990s, solid organs or tissue transplantation have steadily increased, especially in developing areas such as Africa and Asia [1]. Organ transplantation extends life for tens of thousands of individuals. However, infections are common in the general population, so it is inevitable that some persons will die with a potentially transmissible disease and become organ donors. It is accepted in clinical practice that organs are vectors for infectious transmission but are still transplanted [2, 3]. Many of the ‘uncommon’ disease transmissions such as rabies have come with the use of organs from a previously healthy person who subsequently died with an undiagnosed/misdiagnosed neurologic event. Dozens of rabies virus transmissions to recipients via organs or tissues from donors with either undiagnosed or misdiagnosed inflammatory conditions of meningoencephalitis were reported [4–6].

Rabies is an age-old infectious disease, but it remains an important public health problem today. Each year, approximately 59 000 individuals worldwide may die from an infection due to rabies virus, despite the existence of effective prophylaxis [7, 8]. Rabies is a zoonosis, associated with a mortality rate of nearly 100%, caused by neurotropic RNA viruses in the family Rhabdoviridae, genus *Lyssavirus* [7]. Classically, human rabies manifests in either the encephalitic (furious) or paralytic (dumb) form. Diagnosis is extremely difficult in the case of paralytic rabies and when the patient lapses into coma. Human-to-human transmission of rabies has never been confirmed, except extremely rarely as a result of infected organ or tissue transplantation. Undiagnosed or misdiagnosed rabid donors are the reason for rabies virus transmission via solid organ or tissue allotransplantation [9–11]. In 2015 and 2016, two cases of rabies virus transmission via solid organs or tissues allotransplantation (ROTA) were reported in China [6, 12]. In recent years the number of donations and clinical transplants of cornea, bone, skin, heart valve, and other tissues and solid organs increase sharply. In view of this circumstance, there will bound to be more ROTA cases, and we should pay sufficient attention and take appropriate measures to prevent the reoccurrence. In this review, we summary the previous cases, analyze relevant risks and put forward strategies to prevent the occurrence of ROTA.

## The epidemiological characteristics of ROTA

### The pathway of human-to-human transmitted rabies virus

Most cases of rabies are caused by the bite of rabid animals, among which the most common type is the dog, followed by other animals, including cats, ferret badgers, foxes, bats, etc. Rabies virus can infect all mammals, such as cattle, pigs, sheep, wildlife and humans. As a dead-end host of rabies, humans do not typically spread rabies in a human-to-human manner [8, 13–16]. Organ and tissue transplantation is the major way to transmit rabies virus from one human to another, which has been confirmed by laboratory results. The first case of tissue-transplant-related rabies was reported in 1978, and similar events continue to be reported [17]. Once the disease process initiates, it will inevitably lead to the death of the patient, which should focus sufficient attention on ROTA.

### Rabies virus transmission via ROTA occurs worldwide

**Fig. 1:**
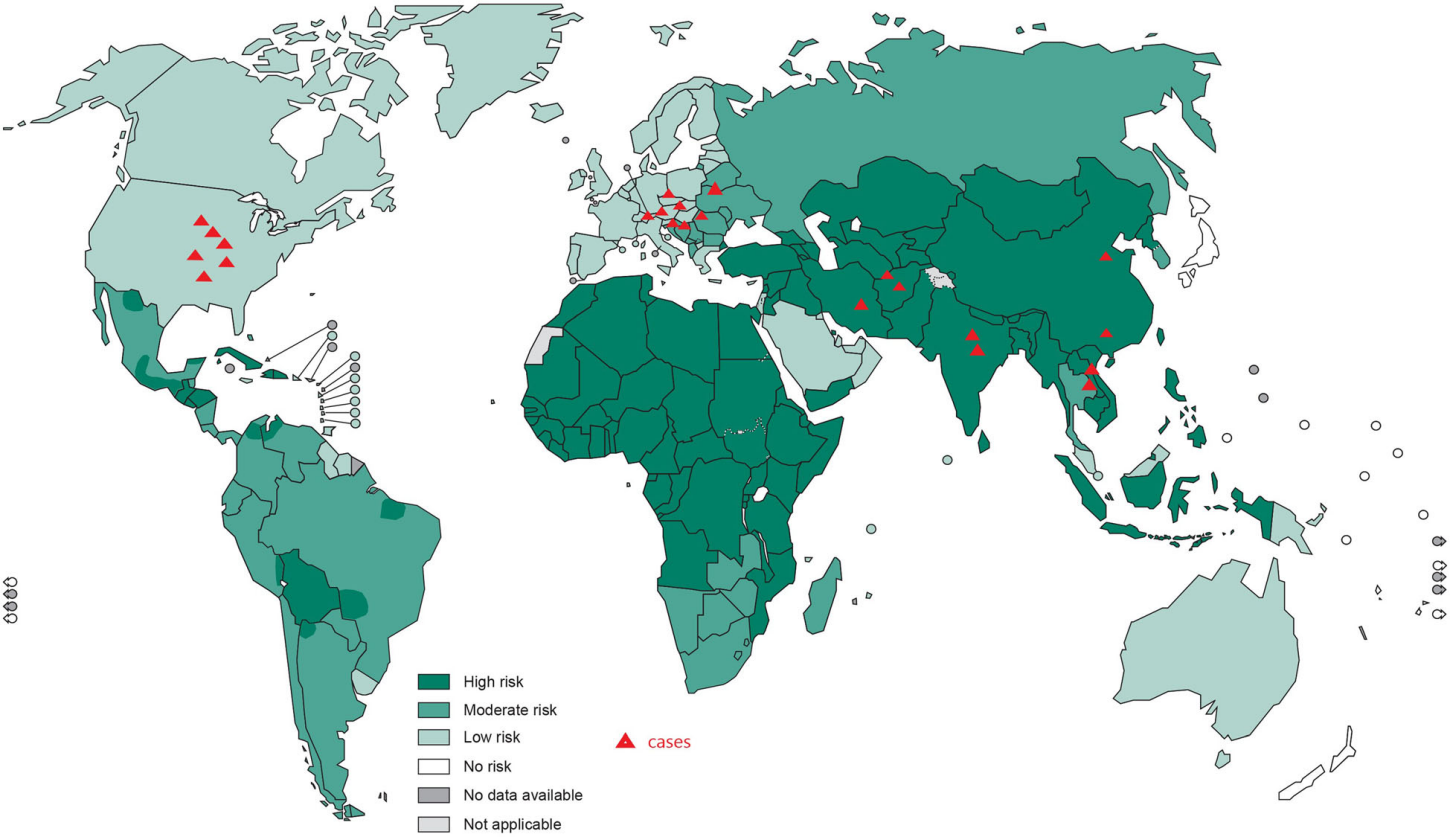
the occurrence of ROTA and the risk of rabies worldwide. The depth of the blue background indicates the incidence risk of rabies, and the red triangle shows the reported cases of ROTA. China and India are high-risk areas with the ROTA report. Europe and the United States are low - risk areas of rabies, however still with ROTA reports. Although the African region is also a high-risk area of rabies without reports of ROTA, sufficient attention should be paid to prevent the occurrence of ROTA, including enhanced surveillance. The background database came from WHO. http://www.who.int/rabies/Global_distribution_risk_humans_contracting_rabies_2013.png?ua=1

Rabies is an infectious disease with a worldwide distribution. In Europe and other developed countries and regions, because of the large-scale use of animal vaccines, the perpetuation and spread of rabies is by wild carnivores and bats, and very few human rabies cases are reported yearly [16, 18]. Rabies remains a very serious illness in poor and developing areas, where it kills locally hundreds or even thousands of people each year [19]. Although organ transplantation requires good medical conditions and involves high surgical costs, an increasing number of people can afford it in various rapidly-developing countries and regions. The first rabies case caused by corneal transplantation in the United States was reported in 1978. Since then, additional cases of ROTA have been reported all over the world. After 2000, ROTA has been reported in countries where more organ transplant surgeries are completed, such as the United States and Germany [20–23]. In 2015 and 2016, two ROTA cases were reported in China [6]. Documented cases of ROTA are summarized in Table 1. To date, at least 13 cases of ROTA have been reported, with infection of 30 recipients overall, among whom 20 patients died and the remaining patients underwent post-exposure prophylaxis (PEP) with survival reported for all but one patient, who was a heart recipient and died of lung infection. The transplanted tissues and organs included the corneas, kidneys, liver, heart, lungs, pancreas, and iliac artery. Similar to rabies, ROTA has a worldwide distribution (Fig. 1). ROTA has been reported in low-risk and high-risk areas; for example, in the United States and Europe, where organ transplant surgery is more prevalent, ROTA continues to be reported, although these are considered as endemic areas with a low risk [24, 25]. Cases of ROTA have begun to be reported in developing countries, such as China, where the number of organ transplant operations is increasing. Because of the weaker surveillance system and a small number of organ transplant surgeries recorded in Africa and other poor regions, there are no reports yet from those areas, but continuous monitoring should be carried out. However, no cases have been reported in Latin America, some measures can exclude patients with fever which is the typical symptom of rabies, mainly because pre-transplant screening for Chagas disease.

**Table 1:** Related reports and basic information of rabies virus transmission via solid organ or tissue allotransplantation

Location	Reported Date	Transplant organ or tissue	Incubation period	Morbidity period	PEP	Outcome	Reference
USA	1978	Corneal	30	12	NO	death	[17]
France	1979	Corneal	33	8	NO	death	[46]
Thailand	1981	Corneal	19	3	NO	death	[42]
	1981	Corneal	31	2	NO	death	
India	1987	Corneal	11	3	NO	death	[28]
India	1988	Corneal	258	5	YES	death	[47]
Iran	1994	Corneal	39	3	NO	death	[48]
Iran	1994	Corneal	25	1	NO	death	
USA	2004	lilac artery	unknown	unknown	NO	death	[10]
		liver	unknown	unknown	NO	death	
		kidney	unknown	unknown	NO	death	
		kidney	unknown	unknown	NO	death	
Germany	2004	lung	unknown	unknown	NO	death	[49, 50]
		kidney	unknown	unknown	NO	death	
		pancreas	unknown	unknown	NO	death	
		corneas	–	–	YES	survival	
		liver	unknown	unknown	NO	survival	
USA	2013	kidney	510	27	NO	death	[5]
		liver	–	–	YES	survival	
		kidney	–	–	YES	survival	
		heart	–	–	YES	survival	
China	2015	kidney	42	46	NO	death	[6]
		kidney	48	34	NO	death	
		Corneal	–	–	YES	survival	
		Corneal	–	–	YES	survival	
China	2016	heart	–	–	NO	death	unpublished
		kidney	41	7	NO	death	
		kidney	45	4	NO	death	
Kuwait	2017	Kidney	105	–	NO	death	[12]
		Kidney	91	–	NO	No diagnosis	
		Corneal	–	–	YES	survival	
		Corneal	–	–	YES	survival	

Note: - means no data reported

### Rabies virus transmission via ROTA occurs in clusters and aggregates with cases

The occurrence of rabies is characterized by aggregation and clustering. This is because a rabid animal may expose more than one person. Molecular biology methods can be used to find identity between the viral variants. ROTA represents the transmission of rabies virus caused by human activities. Organ transplant recipients are a population with a set of obvious medical characteristics. Different tissues and organs from one donor can be transplanted into multiple recipients, resulting in the aggregation of ROTA. In a case reported in Germany in 2004, a donor provided different organs to six recipients, resulting in the death of three patients. In a case reported in the United States in 2013, organs from the one donor were transplanted to four people [5, 26, 27]. Two cases occurred in China in 2015 and 2016, with four patients and three patients, respectively. The other characteristic of ROTA is the nature of the pathogen and the way in which the virus invades the host’s nervous system, i.e., the solid organ and the grafting, instead of the saliva and the bite. It is currently known that corneal transplantation, kidney transplantation, or even vascular transplantation, can cause rabies, and both the organ and the tissue can be used as a potential portal for rabies virus transmission.

### Susceptible population, incubation, and course of ROTA

Humans are susceptible to rabies, but the development of the disease depends on several factors, such as the bite, severity, viral dose, wound care and PEP. Individuals with immune dysfunction or immunodeficiency are more likely to develop infectious disease. A higher incidence of rabies has been observed among organ transplant recipients infected with rabies virus, because of the use of immunosuppressive drugs, which may render them more susceptible to infection. Data from reported cases show that the incubation period of ROTA ranges from 11 days to more than 17 months. The fastest disease course in a case of ROTA occurred in 1987 in India: 11 days from surgery to the death of the patients [28]; whereas the longest corresponded to cases that occurred in 2013 in the United States: the period was 17 months, and another three patients did not develop the disease and survived before using PEP. Patients with rabies usually die within seven to 10 days, while the duration of the disease in ROTA cases varies by organ transplantation of rabies. Once rabies manifests itself clinically, all cases will end in death. The shortest case occurred in 1994 in Iran: the patient died within 1 day. The longest case was reported in China in 2015: the use of sedatives caused a deep coma, and the patient spent 46 days in the intensive care unit (ICU) before dying. Although sedation will extend the course of the disease, once the disease initiates, it leads to death, with very few exceptions. Table 1 also summarizes the case information of ROTA.

### Molecular pathogenesis of ROTA

Rabies is caused by infection with a lyssavirus, such as the type species of the genus, rabies virus, usually through skin wounds or mucous membrane damage. Rabies virus replicates in muscle tissues, followed by invasion of nerve endings and reproduction of a large number of virions in nerve cells; virus is then reverse transmitted to the central nervous system through axons. Numerous copies of the virus spread to the whole nervous system and glands of the host that are innervated by the nervous system, such as the salivary glands. At the late stage of rabies, rabies virus can be observed in glandular cells and epithelial cells. According to clinical manifestations, rabies can be mainly divided into two types: furious and paralytic rabies [7, 8, 13, 29–31].

In organ transplant recipients, it is necessary to use immunosuppressants to prevent rejection of heterologous organs. The use of these drugs reduces the ability of the body to resist foreign pathogens, thus providing an ideal environment for the replication and transmission of pathogens in heterologous organs. There are two main views on the molecular pathogenesis of ROTA. One is that rabies virus is neurotropic: donor organs contain the virus in the nerve endings at the late stage of disease, the virus continues to replicate after transplantation, and via the nervous system, it invades the central nervous system again and causes rabies. The other theory is that the virus exists not only in nerve endings, but also in gland cells and epithelial cells, which represent a reserve of a large amount of the virus in the transplanted organs. When rabies virus enters the recipient, it invades the nervous system to cause rabies in an environment where the host immune system is depressed. Studies have shown that rabies virus from bats can replicate in epithelial cells, which are predominantly paralytic [30]. It is a category III exposure in surgery during transplantation which will contaminate multiple new locations from the surgeon’s hands and instruments, but that’s not the main reason for the spread of the virus.

The detailed molecular mechanism of ROTA is unclear, and the two views described above are consistent with the medical characteristics of rabies virus. The divergence between these two theories is whether the virus carried by the donor exists in nerve terminals or epithelial cells and gland cells. We believe that the two conditions are likely to occur. As a neurotropic virus, rabies virus is rare to replicate in epithelial cells.

Traditionally, rabies virus is a quintessential neurotropic pathogen, with replication and transmission in the nervous system. However, rabies virus undergoes rapid replication and can replicate in selected epithelial or glandular cells. Rabies virus, especially the bat-transmitted type, can be found in epithelial cells, in which its replication and transmission has been confirmed. It has also been reported that significant replication of rabies virus replication occurs in the kidneys. The transplanted organs contain viruses in epithelial cells and nerve terminals, and, under appropriate conditions, rabies virus replicates in many locations and eventually causes the disease [15].

### Reports of rabies virus transmission via ROTA in China

In 2015, a case of ROTA was reported for the first time in China. In Beijing, two patients who received kidney transplantation were diagnosed with rabies after the organ transplantation surgery. The organs received by these two patients were from the same donor, a 6-year-old boy from Guangxi, where about ten cases of rabies are reported per year, thus representing a high-risk rabies region in China. The organ donor had not been diagnosed with rabies, but he appeared to have some symptoms of rabies, such as fever, continuous decline in neurological condition, and progression to coma with progressive loss of all physiological and pathological reflexes. The recipients of the kidney transplants were diagnosed as being exposed to rabies virus using RT-PCR techniques in the laboratory. In 2016, the second case of ROTA was reported in China’s Southern region, which is considered as a high-risk area for rabies. The organ donor was a boy under 2 years old. After treatment in the primary hospital for high fever, coma, and other symptoms, he was transferred to the municipal hospital, where the physician suspected rabies; however, there was a misdiagnosis based on a false negative ELISA test for rabies virus antibody, which is a test with poor sensitivity. Subsequently, his organs were transplanted to three patients, two of whom developed rabies and eventually died. Another recipient died from other complications and was not tested for rabies. ROTA has posed the serious threat to China’s public safety which should be a wake-up call for public health [6, 12].

Rabies is present in China, which is an area that has a high risk of rabies virus infection, as the number of deadly rabies cases per year in this country has been ranked second in the world. In China, the main route of transmission of rabies virus remains a dog bite, accounting for almost 90–95% of cases, followed by wildlife (about 3–5% of cases). Although the association with domestic animals is better appreciated, rabies virus transmission among wildlife is less understood. The prevalence of rabies in China has changed in recent years; the prevalence of rabies in high-incidence areas has decreased, and the occurrence of rabies in low-incidence areas has increased. Rabies remains the third major infectious disease-causing death in China. China will continue to be one of the countries where rabies is rampant for many years to come [18, 32].

Many misdiagnoses have occurred in clinical practice because of the use of sedatives, which conceal the rabies-specific furious phenomenon, thus affecting the diagnosis of rabies. The patients’ personal habits also affect the diagnosis of rabies, such as the situation observed for a case reported in Germany in 2004. Because the organ donor had a history of drug addiction, the clinician believed that the patient’s mental problem was a delusion caused by drug abuse [22]. A clinical diagnosis of rabies should be considered in around one-third of patients with paralytic symptoms. Although the rabies detection network has been established to cover the whole country, China’s vast territory implies that economic development is not balanced; rabies cannot be diagnosed in many areas at present, which has led to the under-diagnosis of this condition. In China, only 1% of rabies cases are confirmed by laboratory tests.

## Measures for the prevention and control of ROTA in the future

### Strengthen publicity and education and improving the diagnosis of rabies

It is necessary to strengthen public and health-care professionals’ awareness of tissue and organ-transplant-related rabies, to improve the screening of organ donors, and to promote the appropriate prophylaxis of donors with suspected rabies for all types of transplant surgery. There should be awareness of the fact that any suspected animal bites are likely to cause rabies, which can also be transmitted through organ transplantation. China remains a country with a serious epidemic of rabies, and organ transplant patients and health-care professionals at all levels should recognize this possibility. Many infectious pathogens can be transmitted through solid organ transplants, e.g., West Nile virus, lymphocytic choriomeningitis virus, Balamuthia mandrill Aris amebae, and HIV [33]. Rabies virus is the only one that can lead to the death of the recipient directly, and there is no effective treatment for this disease. Rabies virus infection should receive more attention in the field of organ transplantation. Concomitantly, it has been proven that timely administration with PEP can prevent and control rabies. In tissue and organ transplant recipients, the use of PEP will also have a preventative effect [28, 34–36].

### Improving the screening of organ donors

Viruses infecting the organ donors are the only source of infection in ROTA. It is necessary to strengthen the screening of donors, with the aim of rejecting solid organs and tissues that contain the virus, thus avoiding ROTA completely. The screening of organ donors should include epidemiological surveys of exposure history and laboratory rabies virus tests.

Rabies has typical clinical symptoms, such as hydrophobia, aerophobia, pharyngeal muscle spasms, and progressive paralysis. Some patients with rabies present mainly with paralysis as the clinical manifestation, accompanied by symptoms of encephalitis, such as fever, headache, vomiting, and irritability. In advanced cases, coma, frequent convulsions, limb muscle flaccidity, and even paralytic symptoms appear. Therefore, donors who exhibit typical rabies symptoms must be screened for virus infection before organ transplantation. If the donor has symptoms of encephalitis, regardless of the presence or absence of exposure to rabies, screening should be performed before transplantation. Donors who have no encephalitis symptoms and for whom epidemiological studies show an absence of recent exposure to suspected sources of infection can be exempted from rabies screening [5, 12, 37, 38].

The screening of organ donors presents many limitations regarding social, biological, and technical aspects. First, in humans, rabies is a rare infectious disease that can be caused by organ transplantation, which is under-detected. There is currently no country or institution that requires the screening of rabies among donors before organ transplantation surgery. Second, during the incubation period, rabies virus infection does not cause symptoms; thus, rabid humans cannot be distinguished from healthy individuals. Third, patients with rabies often use a variety of treatments, including sedatives, which mask the characteristic rabies symptoms, and drug addiction can also lead to a misdiagnosis.

Among the laboratory-based diagnostic methods, RT–PCR is a rapid and sensitive method that has been used widely for the detection of rabies virus nucleic acids. A study reported that in cerebrospinal fluid, saliva, urine, and tear samples collected at different times after the onset of rabies, this method did not detect 100% of rabies virus. With the progress of the disease, the specificity of the detection decreased. In samples of paralytic rabies, the detection rate was lower than that observed for furious rabies, and only one among 14 samples used for testing was positive. In addition, as the presence of rabies virus in biological fluids (such as saliva, spinal fluid, tears and urine) is intermittent, it is necessary to repeatedly collect samples of different types and at different time points. A reliable method to improve the diagnosis of rabies in the laboratory is needed [39, 40].

In China, many patients are in urgent need of organ transplantation. At the same time, there is a very short window of time between the announcement of brain death and transplantation surgery, which implies a limited time for testing the donors. Patients and medical personnel have a very superficial understanding of rabies virus transmission via solid organs or tissues allotransplantation and do not take rabies into account. These factors render ROTA a more serious situation in China.

We strongly appeal for the improvement of screening for rabies among organ donors. We propose the establishment of a more reliable laboratory rabies diagnostic method and the addition of rabies testing to the mandatory list of organ transplant tests, to improve the detection of rabies before organ transplantation and avoid the occurrence of ROTA.

### Suspected cases were protected during surgery

In cases of donors with suspected rabies, an essential biosafety level will be required during the operation. In the course of surgery, medical personnel will come in direct contact with body fluids (such as saliva, blood, and cerebrospinal fluid] of patients with rabies, which contain a large number of copies of virions, and can cause the spread of rabies. Surgical waste must be subjected to high-pressure sterilization procedures to ensure that rabies virus does not pollute the environment [1, 39–41].

### Assess exposure risk and initiate PEP to protect related populations

In the event of ROTA, the appropriate measures should be taken immediately. The main principle is to actively prevent the disease, perform an assessment of personnel who are potentially at risk, and initiate the necessary PEP. In the United States, once a case of rabies has been reported, an epidemiological investigation and intervention by the Center for disease Control and Prevention, the Department of Homeland Security, and the health authorities at all levels is carried out immediately. Risk assessment and preventive measures are conducted, as well as the timely publication of information, to avoid causing social panic. China should learn from this example. The saliva, cerebrospinal fluid, urine, and tears of patients with rabies can contain virus. Contact with body fluids containing rabies virus can also cause transmission. After viral infection, patients should be followed using an acute retrospective epidemiological survey, to identify those who have had close contact with the community staff and relevant health-care workers. According to the exposure level, the risk of rabies among the personnel can be assessed, given the appropriate exposure to prevent the disposal. These steps should also be taken in cases of ROTA. As the recipients of organs from the same infected donor have a high risk of infection, these organs should be discarded immediately and risk assessments and other measures should be taken by contacting the other recipients. The initiation of PEP in patients with organ transplantation is also effective [1]. In cases of immunosuppressed donors (rabies immune globulin [RIG] should be used with vaccine), administration of RIG containing viral neutralizing antibodies, with the earliest such case being reported in 1981 [42, 43]. With the occurrence of corneal transplantation in France, when the donor was discovered to be a rabies patient, the timely removal of the cornea together with administration of RIG and rabies vaccination aided the survival of the recipient [44, 45]. In a case reported in the United States in 2013, the three related recipients received complete PEP and survived. In China, in two cases of corneal transplantation reported in 2015, the recipients received PEP, which resulted in a high level of neutralizing antibodies, and the patient survived. The use of PEP appears effective in cases of ROTA.

## Conclusions

Rabies remains a serious but neglected public health problem all over the world. Together with economic development and the improvement of medical conditions, organ transplantation will save more lives. As a byproduct of organ transplantation, ROTA has begun to appear. In China, there have been two ROTA reports. In combination with the situation in China, ROTA will represent a serious threat to the organ transplant population, which should garner sufficient attention in this field. Canine rabies elimination by mass dog vaccination is still the main way to prevent the happen of rabies in human or ROTA. Currently, more direct measures can be taken to prevent the recurrence of ROTA. With the creation of guidelines relating to the improvement and introduction of new laboratory diagnostic technologies, the strengthening of the screening of organ donors, and the timely administration of PEP, the prevention and control of ROTA will be much improved.

## Additional file

Additional file 1: Multilingual abstracts in the five official working languages of the United Nations. (PDF 198 kb)

## Abbreviations

PEP: Post-exposure prophylaxis
RIG: Rabies immune globulin
ROTA: Rabies virus transmission via solid organs or tissue allotransplantation
