# Supplementary material for: Rabies virus transmission via solid organs or tissue allotransplantation
Source: Infect Dis Poverty. 2018 Aug 15;7:82. doi: 10.1186/s40249-018-0467-7 (PMC6092857; doi:10.1186/s40249-018-0467-7)
Supplement: Supplementary file 1 — Multilingual abstracts in the five official working languages of the United Nations. (PDF 198 kb) [file 40249_2018_467_MOESM1_ESM.pdf]

Translation of the abstract into the five official working languages of the United Nations

انتقال فيروس السعار (داء الكلب) في حالات زراعة الطعم الخيفي لعضو كامل أو لأنسجة

زو- زن لو، وو- يانغ جيو، وجوي- جن وو

الملخص
الخلفية: داء السعار (داء الكلب) هو عبارة عن مرض فيروسي حيواني يمكن أن ينتقل من مريض إلى آخر في حالات زراعة الطعم الخيفي لعضو كامل أو لأنسجة، ويصل معدل الوفيات الناجم عنه إلى 100% تقريباً. وقد سجُلت عشرات الوفيات من جراء انتقال داء السعار (داء الكلب) في حالات زراعة الطعم الخيفي لأعضاء كاملة أو لأنسجة (المعروفة اختصاراً بـROTA) خلال العقود الماضية. ففي عاميّ 2015 و2016، أُبلغ عن حالتين انتقل فيهما فيروس السعار (داء الكلب) عن طريق زراعة الطعم الخيفي لأعضاء كاملة أو لأنسجة في الصين، مما يشدد أكثر وأكثر على مخاطر هذا النوع المميز من داء السعار وأهميته لمرضى زراعة الأعضاء.
النص الرئيسي: في الفترة من العام 1978 إلى العام 2017، تم الإبلاغ عن وجود 13 حالة من انتقال داء السعار (داء الكلب) في حالات زراعة الطعم الخيفي لأعضاء كاملة أو لأنسجة (المعروفة اختصاراً بـROTA)، أسفرت عن عشرات الوفيات في جميع أنحاء العالم، بغض النظر عن كون تلك البلاد أكثر عرضة من غيرها لفيروس السعار أم أقل عرضة من غيرها. تتراوح فترة حضانة فيروس ROTA المسجلة ما بين 11 يوماً إلى أكثر من 17 شهراً، في حين أنه يُعتقد بوجه عام أن فترة حضانة داء السعار (داء الكلب) الأصلية تتراوح ما بين قرابة الأسبوع إلى عدة أعوام. صحيح أن طريقة نشوء مرض ROTA ليست واضحة، إلا أن اللجوء إلى العلاج الوقائي بعد التعرض للفيروس (PEP) من الممكن أن يكون له دور في وقاية مرضى زراعة الأعضاء. بالإضافة إلى ذلك، فقد لخصنا في هذه الدراسة تقارير عن فيروس ROTA في الصين، وقرنّاها بالموقف الفعلي لأعمال رصد داء السعار (داء الكلب) والقضاء عليه، واقترحنا عدداً من التدابير المضادة للوقاية من فيروس ROTA والسيطرة عليه مستقبلاً.
الاستنتاج: لا شك أن فهم أهمية فيروس ROTA، وفحص الأعضاء المشتبه بإصابتها بالفيروس، وتقييم المخاطر، ووقاية الفئة المعنية من السكان من شأنه أن يشكّل وسيلة ناجعة للوقاية من الفيروس والسيطرة عليه لمنع انتشاره أكثر من ذلك.

Translated from English version into Arabic by Heba Kandel and Free bird, through

TRANSLATORS WITHOUT BORDERS

器官移植引发狂犬病

Xue-Xin Lu, Wu-Yang Zhu and Gui-Zhen Wu

摘要
引言：狂犬病是一种致死率几乎为 100%的人兽急性传染病，并可以通过器官或组织移植的途径传播。在过去的几十年中，已经有多例因器官移植引起狂犬病的病例被记录。在 2015 年和 2016 年，中国报道了两例因为器官移植而引起的狂犬病，促使了对器官移植引起狂犬病的风险和重要性的认识。
正文：从 1978 年至 2017 年，世界范围内至少有 13 例器官移植引发狂犬病的报道，并造成了数十人了的死亡。无论是狂犬病的高危或低危地区，器官移植引起的狂犬病均有发生，其潜伏期最短为 11 天，最长的超过了 17 个月。这种狂犬病的发病机制还未被阐明，但实施暴

露后处置可以对器官受体提供保护作用。本文总结了器官移植引发狂犬病的相关报道，结合中国在狂犬病监测和消除方面的现状，对预防器官移植引起狂犬病提出了相应的建议。
结论：了解器官移植引发狂犬病的重要性，对可疑的器官供体进行筛查，评估相关人群的风险并及时进行暴露后处置是预防这一类狂犬病发生的重要措施。

Translated from English version into Arabic by Xue-Xin Lu

Transmission du virus de la rage par allogreffe d'organes solides ou de tissus

Xue-Xin Lu, Wu-Yang Zhu et Gui-Zhen Wu

Résumé
Contexte : La rage, dont le taux de mortalité avoisine 100 %, est une maladie virale zoonotique pouvant être transmise par allogreffe d'organes solides ou de tissus. Des dizaines de décès dus à la rage transmise par allogreffe d'organes solides ou de tissus (ROTA) ont été documentés au cours des dernières décennies. En 2015 et 2016, deux cas de transmission du virus par allogreffe de tissus ou d'organes solides ont été signalés en Chine, soulignant d'autant plus le risque et l'importance de ce type particulier de transmission de la rage pour les receveurs d'organes.
Corps du texte : De 1978 à 2017, au moins 13 cas de ROTA, responsables de dizaines de décès, ont été signalés à travers le monde, dans les pays à risque élevé comme ceux à faible risque de rage. La période d'incubation rapportée pour la ROTA varie de 11 jours à plus de 17 mois, tandis que celle typiquement associée à la rage est généralement comprise entre une semaine et plusieurs années. La pathogenèse de la ROTA n'est pas clairement définie, mais l'utilisation de prophylaxie post-exposition (PEP) peut jouer un rôle protecteur chez les receveurs de greffe. Nous présentons également un résumé des rapports sur la ROTA en Chine associé à un état de la situation quant aux efforts de surveillance et d'élimination de la rage. Nous proposons pour l'avenir des mesures de prévention et de contrôle de la ROTA.
Conclusion : Comprendre l'importance de la ROTA, le dépistage des organes suspectés, l'évaluation du risque et la protection de la population concernée seront des moyens efficaces de prévenir et contrôler l'apparition d'autres cas de ROTA.

Translated from English version into French by Lina Scarpellini and Fane Wann, through

TRANSLATORS
WITHOUT BORDERS

Бешенство — передача вируса посредством аллотрансплантации паренхиматозных органов или тканей

Сюэ-Синь Лу, У-Ян Чжу и Гуй-Чжэнь У

Аннотация

Справочная информация: Бешенство, уровень смертности от которого составляет почти 100%, является зоонозным заболеванием вирусной природы и способно передаваться посредством аллотрансплантации паренхиматозных органов или тканей. В течение последних десятилетий были задокументированы десятки смертей от бешенства, спровоцированного аллотрансплантацией паренхиматозных органов или тканей. В 2015 и 2016 годах в Китае были зарегистрированы два случая передачи вируса бешенства посредством аллотрансплантации паренхиматозных органов или тканей, что ещё раз подчёркивает как опасность, так и значительность указанного особого вида бешенства среди реципиентов при трансплантации органов.

Основной текст: В период с 1978 года по 2017 год по всему миру, включая страны как с высоким, так и с низким уровнем риска по данному заболеванию, были зарегистрированы по меньшей мере 13 случаев бешенства, спровоцированного аллотрансплантацией паренхиматозных органов или тканей, что привело к десяткам смертей. Зарегистрированный инкубационный период бешенства, спровоцированного аллотрансплантацией паренхиматозных органов или тканей, колеблется от 11 дней до более 17 месяцев, тогда как исторически инкубационным периодом бешенства обычно считается промежуток от приблизительно 1-й недели до нескольких лет. Патогенез бешенства, спровоцированного аллотрансплантацией паренхиматозных органов или тканей, пока неясен, однако использование постконтактной профилактики (ПКП) среди реципиентов при трансплантации способно выполнять защитную функцию. Также нами обобщаются выводы по бешенству, спровоцированному аллотрансплантацией паренхиматозных органов или тканей в Китае, в сочетании с фактическим положением в отношении деятельности по контролю и искоренению бешенства, кроме того, нами предлагаются меры противодействия по профилактике и контролю над указанным видом заболевания в будущем.

Выводы: Понимание значительности бешенства, спровоцированного аллотрансплантацией паренхиматозных органов или тканей, обследование органов с подозрением на присутствие данного заболевания, оценка риска, а также защита задействованного населения станут эффективными методами профилактики и контроля в случаях дальнейшего возникновения бешенства, спровоцированного аллотрансплантацией паренхиматозных органов или тканей.

Translated from English version into Russian by Liudmila Tomanek and Margarita, through

TRANSLATORS
WITHOUT BORDERS

Transmisión del virus de la rabia por medio del alotrasplante de vísceras macizas y tejidos

Xue-Xin Lu, Wu-Yang Zhu y Gui-Zhen Wu

Resumen

Antecedentes: La rabia, cuya tasa de mortalidad es casi del 100 %, es una enfermedad zoonótica

vírica que puede transmitirse a través del alotrasplante de vísceras macizas o tejidos. Durante las últimas décadas, se han documentado docenas de muertes por la rabia transmitida a través del alotrasplante de vísceras macizas y tejidos (RAOT). En 2015 y 2016, se recogieron en China dos casos de transmisión del virus de la rabia a través del alotrasplante de vísceras macizas o tejidos, lo que destaca aún más el riesgo y la importancia de este tipo especial de rabia para receptores de trasplantes de órganos.

Texto principal: Desde 1978 a 2017, se han documentado por todo el mundo al menos 13 casos de RAOT, que causaron docenas de muertes, tanto en los países de alto riesgo como de bajo riesgo de contagio de rabia. El periodo de incubación descrito de la RAOT oscila entre los 11 días y más de 17 meses, mientras que el periodo de incubación histórica de la rabia se considera que oscila generalmente entre ~1 semana y varios años. La patogénesis de la RAOT no está muy clara, pero el uso de la profilaxis posexposición (PPE) puede desempeñar un papel protector en los receptores de trasplantes.   También resumimos informes sobre la RAOT en China, así como la situación real respecto al trabajo realizado sobre la vigilancia y eliminación de la rabia y sugerimos medidas para la prevención y control de la RAOT en el futuro.

Conclusiones: Entender la importancia de la RAOT, examinar los órganos sospechosos, evaluar el riesgo y proteger a la población relacionada será una manera eficaz para prevenir y controlar más casos de RAOT.

Translated from English version into Spanish by Manuela Evans and Noelia Bernárdez, through

TRANSLATORS
WITHOUT BORDERS
